# Characterisation of microbial communities within aggressive prostate cancer tissues

**DOI:** 10.1186/s13027-016-0112-7

**Published:** 2017-01-13

**Authors:** Melissa A. Yow, Sepehr N. Tabrizi, Gianluca Severi, Damien M. Bolton, John Pedersen, Australian Prostate Cancer BioResource, Graham G. Giles, Melissa C. Southey

**Affiliations:** 1 Genetic Epidemiology Laboratory, Department of Pathology, Faculty of Medicine, Dentistry and Health Sciences, University of Melbourne, VIC, Australia 3010; 2 Department of Microbiology and Infectious Diseases, Royal Women’s Hospital, Parkville, VIC Australia 3052; 3 Department of Obstetrics and Gynaecology, University of Melbourne, Parkville, VIC Australia 3010; 4 Murdoch Childrens Research Institute, Parkville, VIC Australia 3052; 5 Cancer Epidemiology and Intelligence Division, Cancer Epidemiology Centre, Cancer Council Victoria, Level 2, 615 St Kilda Road, Melbourne, VIC Australia 3004; 6 Centre for Epidemiology and Biostatistics, Melbourne School of Population and Global Health, University of Melbourne, Level 3, 207 Bouverie Street, Carlton, VIC Australia 3053; 7 Department of Surgery, University of Melbourne, Austin Health, 145 Studley Road, Heidelberg, VIC Australia 3084; 8 TissuPath, 92-96 Ricketts Road, Mount Waverley, VIC Australia 3149; 9 Human Genetics Foundation (HuGeF), Via Nizza, 52-10126 Torino, Italy; 10 Australian Prostate Cancer BioResource, the Prostate Cancer Research Program, Department of Anatomy and Developmental Biology, Monash University, Melbourne, VIC Australia 3800

**Keywords:** Prostate cancer, Sexually transmitted infection, Infection, 16S rRNA, RNA, cDNA, *Propionibacterium acnes*

## Abstract

**Background:**

An infectious aetiology for prostate cancer has been conjectured for decades but the evidence gained from questionnaire-based and sero-epidemiological studies is weak and inconsistent, and a causal association with any infectious agent is not established. We describe and evaluate the application of new technology to detect bacterial and viral agents in high-grade prostate cancer tissues. The potential of targeted 16S rRNA gene sequencing and total RNA sequencing was evaluated in terms of its utility to characterise microbial communities within high-grade prostate tumours.

**Methods:**

Two different Massively Parallel Sequencing (MPS) approaches were applied. First, to capture and enrich for possible bacterial species, targeted-MPS of the V2-V3 hypervariable regions of the 16S rRNA gene was performed on DNA extracted from 20 snap-frozen prostate tissue cores from ten “aggressive” prostate cancer cases. Second, total RNA extracted from the same prostate tissue samples was also sequenced to capture the sequence profile of both bacterial and viral transcripts present.

**Results:**

Overall, 16S rRNA sequencing identified *Enterobacteriaceae* species common to all samples and *P. acnes* in 95% of analyzed samples. Total RNA sequencing detected endogenous retroviruses providing proof of concept but there was no evidence of bacterial or viral transcripts suggesting active infection, although it does not rule out a previous ‘hit and run’ scenario.

**Conclusions:**

As these new investigative methods and protocols become more refined, MPS approaches may be found to have significant utility in identifying potential pathogens involved in disease aetiology. Further studies, specifically designed to detect associations between the disease phenotype and aetiological agents, are required.

**Electronic supplementary material:**

The online version of this article (doi:10.1186/s13027-016-0112-7) contains supplementary material, which is available to authorized users.

## Background

First proposed in the early 1950s, an infectious aetiology for prostate cancer has since been widely investigated using conventional and serology-based case–control designs and some cohort studies but the evidence from these has been generally weak and inconsistent. A causal association is yet to be established.

Recent support for a role of infection in prostate cancer risk came from the detection of a novel candidate, *Propionibacterium acnes*, within prostate cancer tissues [1, 2]. There is also evidence of association between prostate cancer risk and gene variants of COX-2 [3], RNASEL [4] and TLR4 [5], identified in cases of hereditary prostate cancer, indicating that infection and the host response to infection may be involved in the development of prostate cancer.

Studies that have investigated the role of infectious agents in the aetiology of prostate cancer have adopted single organism targeted approaches or have identified microbial constituents based on amplification of various hypervariable regions of the 16S rRNA gene in concert with traditional cloning and sequencing methods [6–9]. Single organism targeted approaches are limited by their specificity while traditional broad-range 16S rRNA gene amplification, cloning and Sanger sequencing can be laborious and costly, depending on the scale of the study, number and complexity of samples. When compared with conventional sequencing methods, cyclic array-based massively parallel sequencing (MPS) methods, albeit with shorter read length capability and less accuracy in base calling, offer efficiencies in terms of cost, time and scalability.

The principal hypothesis that guided the direction of the work presented in this study was that persistent, rather than transient, infection of the prostate gland by a sexually transmitted or other infectious agent would be associated with risk. Thus, evidence of infection at the tissue level was sought by utilising two different molecular approaches, targeted partial 16S rRNA gene sequencing and total RNA sequencing using MPS. The overall objective of this study was to investigate the presence of infectious agent(s) in histopathologically determined aggressive prostate cancer cases (Gleason score ≥ 8).

## Methods

### Samples

Fresh-frozen scalpel-excised prostate tissue from males that had undergone radical prostatectomy with a Gleason score of ≥ 8 and tumour stage ranging from pT2c to pT3b (inclusive) were obtained from the Australian Prostate Cancer Bioresource [10] (*n* = 10). Tumour and benign tissues were provided for each case and the presence/absence of malignant tissue was confirmed by histopathology by a single pathologist (JP).

### Nucleic acid extraction

Frozen tissue was disrupted by freeze fracture, Buffer RLT Plus (Qiagen, Hilden, Germany) containing β-mercaptoethanol was added. The lysate was further homogenised using a QIAshredder® (Qiagen, Hilden, Germany) column and then underwent enzymatic digestion and nucleic acid extraction with the AllPrep DNA/RNA Mini Kit (Qiagen, Hilden, Germany) according to the manufacturer’s instructions. Both DNA and RNA isolates were stored at −80 °C (Additional file 1).

### Quantitative and qualitative assessment of extracted DNA and RNA

The concentration and integrity of sample RNA was assessed with the Bioanalyzer 2100 instrument (Agilent Technologies) using RNA 6000 Nano Kit (Agilent Technologies). The concentration of sample DNA was assessed by Qubit® 1.0 Fluorometer (Life Technologies, Carlsbad, California, USA) and the Qubit® dsDNA BR Assay Kit (Life Technologies, Carlsbad, California, USA).

### Quantification, normalisation and pooling of libraries

Each RNA sample was normalised to 100 μg/μL in UltraPure™ DNAse/RNAse-Free Distilled Water (Invitrogen™, Burlington, USA). Normalised RNA samples were pooled in equimolar amounts according to tissue type i.e. “malignant” or “benign”.

### 16S rRNA amplicon sequencing

#### 16S rRNA polymerase chain reaction

Each PCR reaction contained 1X GeneAmp® PCR Buffer II (Roche Molecular Systems, Inc. Branchburg Township, USA), 10 μM (each) forward and reverse primer, 0.1 U AmpliTaq Gold® DNA polymerase (ThermoFisher Scientific, Waltham, Massachusetts, USA), 2.5 μM MgCl_2_, 400 μM dNTPs, 2 μL of template DNA in a final volume of 20 μL with UltraPure™ DNAse/RNAse-Free Distilled Water (Invitrogen™, Burlington, USA). Amplification of each sample was performed in triplicate using a Veriti® 96-well Thermal Cycler (Applied Biosystems, Forster City, CA, USA). Negative amplification controls included dH_2_O and TE buffer and the positive amplification control was *Salmonella typhimurium* (0.5 ng/μL). Cycling conditions were as follows: 95 °C for 5 min, 35 cycles at 95 °C for 45 s, 56 °C for 60 s and 72 °C for 90 s, with a final extension at 72 °C for five minutes.

#### Primers

Universal primers 101F/534R and 515F/806R were used to amplify the V2-V3 and V4 hypervariable region of the 16S rRNA gene, respectively. The V4 region primer constructs were taken from Caporaso et al. (2011) [11] (supplementary methods). The V2-V3 region primer constructs were modified from [11] using V2-V3 region specific primers [12] to target the 16S rRNA V2-V3 hypervariable region (Additional file 1). Reverse primers were barcoded to enable multiplexing of samples.

#### Purification of PCR products

Replicate wells were combined for each sample and excess primers, primer dimers and extraneous products were removed using a double-sided size selection/clean-up with Agencourt® AMPure® XP beads (Beckman Coulter, Inc., Massachusetts, U.S.A). Purified product was eluted in 30 μL dH_2_O.

### Quantification and normalization of library pools

Library size and quantity were assessed using the Bioanalyzer 2100 using the High Sensitivity DNA kit (Agilent Technologies Inc., Waldbronn, Germany). Individual samples were combined in equimolar quantities for sequencing.

#### Sequencing

Three custom primers were used for sequencing of the 16S rRNA V4 region amplicons as described in [11] and the 16S rRNA V2-V3 region amplicons as adapted from Caporaso et al. (2011) [11]. The libraries were sequenced by using the MiSeq® 500 cycle Reagent Kit v2 (Illumina, Inc., San Diego, CA, USA).

#### Data analysis

The quality of raw reads was assessed using FastQC v0.10.1 [13]. Paired-end reads were then stitched using FLASh (Fast Length Adjustment of Short reads) v1.2.6 [14] to generate full length reads of the of the sequenced amplicons. The quality of the FLASh-stitched reads were again assessed using FastQC v0.10.1 [13].

The QIIME (Quantitative Insights Into Microbial Ecology) pipeline and software package (version 1.7.0) [15] were used for data analyses using Closed-reference Operational Taxonomic Unit (OTU) picking. The sequences were clustered against a reference sequence collection [16] (Greengenes 12_10 reference collection) and any reads that did not hit a sequence at 97% sequence similarity to the reference sequence collection were excluded from downstream analysis.

### Total RNA/cDNA sequencing

#### Library preparation and sequencing

Library preparation was performed using the Illumina® TruSeq® Stranded Total RNA Sample Preparation Kit in accordance with the manufacturer’s instructions, however, did not include the initial poly(A) purification step (supplementary methods). The libraries were assessed with the Bioanalyser 2100 using the Bioanalyser DNA 1000 kit (Agilent). Individual libraries (tumour and cancer-unaffected prostate pools) were normalised to 2 nM. Sequencing was performed on the HiSeq™ 2000.

#### Data analysis

Raw data underwent quality control and sequencing adapters were removed using Nesoni [17]. The full data set was queried for specific viral genomes (including human papillomaviruses 16 and 18, Herpes simplex virus 2 and Polyomaviruses) using human endogenous retroviruses (HERVs) as internal control as HERVs are remnant ancient retroviral sequences integrated into human germline DNA, some of which are actively transcribed. Reads were mapped to human rRNA (and other non-coding RNA) and to human mRNA using the SHort Read Mapping Package (SHRiMP) [18] and Burrows-Wheeler Aligner (BWA) [19], respectively. Aligned reads were removed from the dataset. Unmapped reads were assembled into contiguous sequences using the *de novo* assembler Velvet [20], under kmer values of 55, 65, 75 and 85. The assemblies were queried with Easy-Web-BLAST+ [21] for 16S rRNA sequences and the presence of viral proteins (specifically all viral polymerases within the NCBI’s RefSeq viral protein reference database [22]).

## Results

### Characteristics of the case series

The mean age at radical prostatectomy of patients was 64.5 years. Three cases underwent radical laparoscopic robotic prostatectomy while the remaining seven cases had open radical retropubic prostatectomy. All cases were considered to be of an aggressive nature and were selected on the basis of a Gleason score of ≥ 8 and a TNM stage of at least PT2c (Table 1).

**Table 1:** Histopathological features (Gleason score and TNM stage), age at radical prostatectomy and pre-operative PSA (ng/μL) for ten prostate cancer cases obtained from the Australian Prostate Cancer BioResource

Patient ID	Gleason Score	TNM Stage	Age (years) at resection	Surgical type	Pre-operative PSA (ng/μL)
PI	8	PT3AN0	67.6	Open	26.7
P2	9	PT3B	68.9	Open	6.2
P3	9	PT3AN1MX	73.3	Open	1.9
P4	9	PT2CN0	61.5	Open	3.1
P5	9	PT2C	59.2	Robot	5.7
P6	9	PT3BN0	64.4	Robot	n/a
P7	8	PT3AN0	68.1	Open	13.9
P8	9	PT3A	61.1	Open	9.2
P9	9	PT3AN0	53.4	Open	n/a
P10	8	PT3AN0	67.8	Robot	8.8

### 16S rRNA V4 hypervariable region

One thousand three hundred and twenty four unique OTUs were identified in all 20 prostate tissue samples combined. Per sample, the mean number of OTUs present was 231.55 (SD 48.45) and ranged from 151 to 314. Community composition was reasonably complex.

Overall, the most abundant taxa identified were assigned to the family *Enterobacteriaceae* (70.1%) and the genus *Escherichia* (6.9%). There were five other unique OTUs that represented ≥ 1% of the microbial community observed across all samples. These taxa included *Pseudomonadaceae* (1.2%), *Comamonadaceae* (1.2%), *Ralstonia* (1.7%), *Pseudomonas* (1.3%) and *Acinetobacter* (1.1%). There were five OTUs that represented 0.5 < 1% of the microbial community observed and these included *Corynebacterium* (0.8%), *Caulobacteriaceae* (0.7%), *Curvibacter* (0.7%) *Aerococcus* (0.6%) and *Bradyrhizobium* (0.6%) The remaining 13.7% of sequences were assigned to 308 other unique OTUs (Additional file 2).

The greatest proportion of sequences, ranging from 37.2 to 81.2%, for each individual sample was represented by the family *Enterobacteriaceae*.. The prevalence of *Escherichia* ranged from 3.1 to 10.3% in the samples. Both taxa were represented in every sample. While there was up to a two-fold difference in the number of observed OTUs (151 to 314) among samples, the community composition of the most abundant samples (abundance > 0.5%) was reasonably consistent across individual samples, however, some taxa including *Pseudomonadaceae, Aerococcus*, *Corynebacterium* and *Actinobacter lwoffii* were overrepresented in a number of samples when compared to their contribution to overall abundance (Additional file 2).

**Fig. 1:**
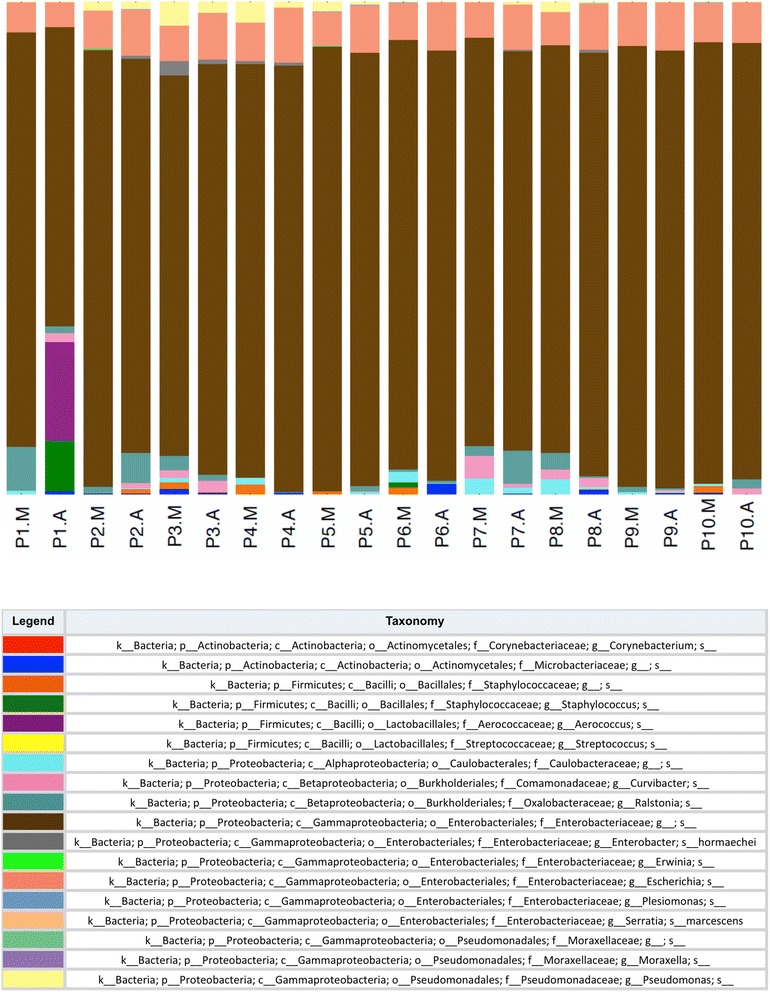
Taxa summary of ‘core’ OTUs identified in 95% of samples (*n* = 20) that underwent sequencing of the V4 hypervariable region of the 16S rRNA gene. The figure depicts the relative contribution of each member of the ‘core’ community to each sample in addition to its overall contribution to the core community over all samples combined. The contribution of taxa to the core community is expressed as a percentage. The letter A next to the patient ID denotes “adjacent” tissue and M denotes “malignant” tissue. The letters in the taxonomy column refer to k – kingdom, p – phylum, c –class, o –order, f – family, g – genus, s – species

A group of 18 OTUs was found to be present in 95% of samples (Table 2). While these 18 OTUs only represented a small proportion (on average 7.8%) of the overall membership of prostatic microbial community, they contributed to a large proportion (84.6%) of the relative abundance of the total communities of the 20 samples sequenced. The relative contribution of each ‘core’ OTU was reasonably consistent across samples (Fig. 1) with *Enterobacteriacae* (84.4%) and *Escherichia* (8.3%) the most abundant taxa contributing the ‘core’ community.

**Table 2:** Taxonomic assignments of the 18 OTUs present in 95% of samples (*n* = 20) that underwent sequencing of the V4 hypervariable region of the 16S rRNA gene and their relative abundance

Core OTUs shared by 95% of samples	Relative abundance of OTU within the total community
	%
k_Bacteria; p_Proteobacteria; c_Gammaproteobacteria; o_Enterobacteriales; f_Enterobacteriaceae; g_; s_	70.1%
k_Bacteria; p_Proteobacteria; c_Gammaproteobacteria; o_Enterobacteriales; f_Enterobacteriaceae; g_Escherichia; s_	6.9%
k_Bacteria; p_Proteobacteria; c_Betaproteobacteria; o_Burkholderiales; f_Oxalobacteraceae; g_Ralstonia; s_	1.7%
k_Bacteria; p_Proteobacteria; c_Gammaproteobacteria; o_Pseudomonadales; f_Pseudomonadaceae; g_Pseudomonas; s_	1.3%
k_Bacteria; p_Actinobacteria; c_Actinobacteria; o_Actinomycetales; f_Corynebacteriaceae; g_Corynebacterium; s_	0.8%
k_Bacteria; p_Proteobacteria; c_Betaproteobacteria; o_Burkholderiales; f_Comamonadaceae; g_Curvibacter; s_	0.7%
k_Bacteria; p_Proteobacteria; c_Alphaproteobacteria; o_Caulobacterales; f_Caulobacteraceae; g_; s_	0.7%
k_Bacteria; p_Firmicutes; c_Bacilli; o_Lactobacillales; f_Aerococcaceae; g_Aerococcus; s_	0.6%
k_Bacteria; p_Firmicutes; c_Bacilli; o_Bacillales; f_Staphylococcaceae; g_Staphylococcus; s_	0.4%
k_Bacteria; p_Actinobacteria; c_Actinobacteria; o_Actinomycetales; f_Microbacteriaceae; g_; s_	0.4%
k_Bacteria; p_Firmicutes; c_Bacilli; o_Bacillales; f_Staphylococcaceae; g_; s_	0.3%
k_Bacteria; p_Proteobacteria; c_Gammaproteobacteria; o_Enterobacteriales; f_Enterobacteriaceae; g_Enterobacter; s_hormaechei	0.3%
k_Bacteria; p_Proteobacteria; c_Gammaproteobacteria; o_Pseudomonadales; f_Moraxellaceae; g_; s_	0.2%
k_Bacteria; p_Proteobacteria; c_Gammaproteobacteria; o_Enterobacteriales; f_Enterobacteriaceae; g_Plesiomonas; s_	0.1%
k_Bacteria; p_Firmicutes; c_Bacilli; o_Lactobacillales; f_Streptococcaceae; g_Streptococcus; s_	0.1%
k_Bacteria; p_Proteobacteria; c_Gammaproteobacteria; o_Enterobacteriales; f_Enterobacteriaceae; g_Erwinia; s_	<0.1%
k_Bacteria; p_Proteobacteria; c_Gammaproteobacteria; o_Enterobacteriales; f_Enterobacteriaceae; g_Serratia; s_marcescens	<0.1%
k_Bacteria; p_Proteobacteria; c_Gammaproteobacteria; o_Pseudomonadales; f_Moraxellaceae; g_Moraxella; s_	<0.1%
Sum of “core” OTUs (across 95% of samples)	84.6%

The letters in the taxonomy column refer to k – kingdom, p – phylum, c –class, o –order, f – family, g – genus, s – species

### 16S rRNA V2-V3 hypervariable region

Six hundred and thirty four unique OTUs were present in all 20 prostate tissue samples combined. On a per sample basis, the mean number of OTUs present was 117.95 (SD 23.95) and ranged from 71 to 160.

All samples combined, *Enterobacteriaceae* was dominant taxon (55.4%), followed by *Escherichia* (20.9%). There were seven additional OTUs with an abundance ≥ 1% including *Comamonadaceae* (1.8%), *Hyphomonadaceae* (1.5%), *Pseudomonas* (3.4%), *Corynebacterium* (1.3%), *Tepidimonas* (1.2%), *P. acnes* (1.1%) and *Acinetobacter* (1.0%). *Ralstonia* and *Lutemonas* represented 0.8 and 0.6% of the total microbial community, respectively. The remaining 11% of sequences comprised of the 194 other OTUs (Additional file 3).

The highest proportion of sequences for each individual sample was assigned to *Enterobacteriaceae* with an abundance ranging from 21.9 to 69.4% followed by *Escherichia* with an abundance ranging from 6.5 to 29.9%. Both were represented in every sample. The contribution of the most abundant taxa (>0.5%) to the community composition of each sample was reasonably consistent despite a two-fold difference in the number of observed OTUs (71 to 160). However, some taxa were overrepresented in a number of samples when compared to their contribution to overall abundance (Additional file 3).

**Fig. 2:**
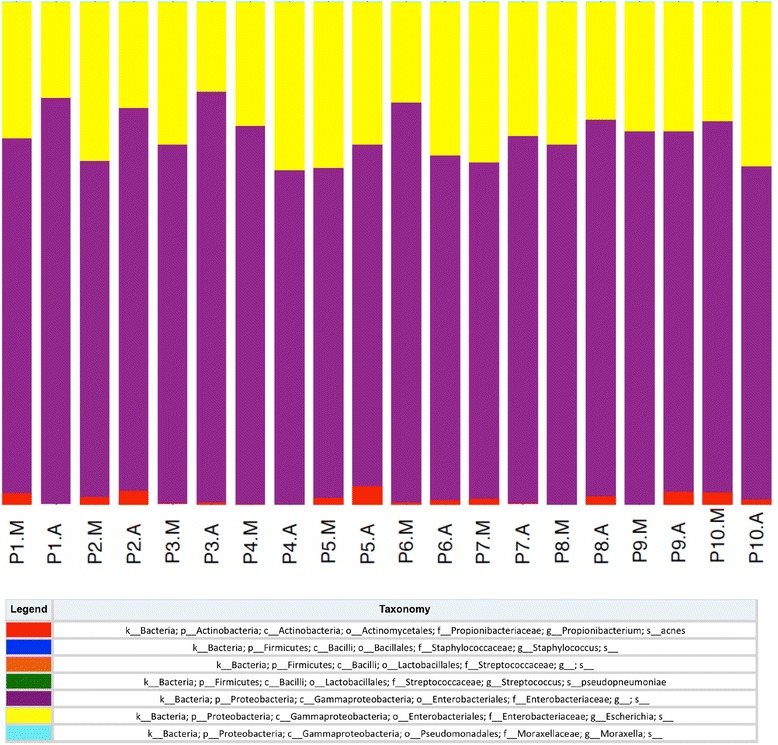
Taxa summary of ‘core’ OTUs identified in 95% of samples (*n* = 20) that underwent sequencing of the V2-V3 region of the 16S rRNA gene. The figure depicts the relative contribution of each member of the ‘core’ community to each sample in addition to its overall contribution to the core community over all samples combined. The contribution of taxa to the core community is expressed as a percentage. The letter A next to the patient ID denotes “adjacent” tissue and M denotes “malignant” tissue. The letters in the taxonomy column refer to k – kingdom, p – phylum, c –class, o –order, f – family, g – genus, s – species

Seven OTUs were represented in 95% of samples (*n* = 20) and together they constituted the ‘core’ community within these prostate tissue samples (Table 3). These OTUs were assigned to *Enterobacteriaceae* and *Streptococcaceae*, *Staphylococcus*, *Escherichia*, *Moraxella*, *Propionibacterium acnes* and *Streptococcus pseudopneumoniae*. Despite these ‘core’ OTUs representing only a small proportion (on average 5.9%) of the mean number of OTUs that comprise the overall prostatic microbial community, they contributed to a very large proportion (77.9%) of the relative abundance of the total communities of the 20 samples sequenced. The relative contribution of each of the seven ‘core’ OTUs was reasonably consistent across individual samples (Fig. 2). *Enterobacteriaceae* and *Escherichia* were observed to be the most abundant taxa contributing to the ‘core’ community with a relative abundance of 72.2 and 26.6% respectively.

**Table 3:** Taxonomic assignments of the 7 OTUs present in 95% of samples (*n* = 20) that underwent sequencing of the V2-V3 region of the 16S rRNA gene and their relative abundance

Core OTUs shared by 95% of samples	Relative abundance of OTU within the total community
	%
k_Bacteria; p_Proteobacteria; c_Gammaproteobacteria; o_Enterobacteriales; f_Enterobacteriaceae; g_; s_	55.4%
k_Bacteria; p_Proteobacteria; c_Gammaproteobacteria; o_Enterobacteriales; f_Enterobacteriaceae; g_Escherichia; s_	20.9%
k_Bacteria; p_Actinobacteria; c_Actinobacteria; o_Actinomycetales; f_Propionibacteriaceae; g_Propionibacterium; s_acnes	1.1%
k_Bacteria; p_Firmicutes; c_Bacilli; o_Bacillales; f_Staphylococcaceae; g_Staphylococcus; s_	0.4%
k_Bacteria; p_Firmicutes; c_Bacilli; o_Lactobacillales; f_Streptococcaceae; g_; s_	0.1%
k_Bacteria; p_Firmicutes; c_Bacilli; o_Lactobacillales; f_Streptococcaceae; g_Streptococcus; s_pseudopneumoniae	<0.1%
k_Bacteria; p_Proteobacteria; c_Gammaproteobacteria; o_Pseudomonadales; f_Moraxellaceae; g_Moraxella; s_	<0.1%
Sum of “core” OTUs (across 95% of samples)	77.90%

The letters in the taxonomy column refer to k – kingdom, p – phylum, c –class, o –order, f – family, g – genus, s – species

### Total RNA sequencing

Human endogenous retroviral sequences (HERVs) were successfully detected in both benign and malignant datasets. After removing human ribosomal RNA and other non-coding read pairs, approximately 20 million read pairs remained for each of the malignant and benign prostate tissue datasets. Removing human mRNA left approximately 2.8 million unmapped read pairs for both the malignant and benign datasets. The unmapped reads were assembled into contiguous sequences using Velvet at kmer values of 55, 65, 75 and 85 and were queried for sequences of interest using BLAST. Sequences identified as belonging to *Pseudomonas* spp. were detected in the benign prostate tissue dataset. No sequences analogous to the NCBI RefSeq [22] library of viral polymerases (with the exception of HERVs) were detected. No specific viral sequences including human papillomaviruses, polyomaviruses, herpes simplex virus 1 and 2, were detected in either dataset.

## Discussion

We used broad-range methods (one targeted and one agnostic) to explore and characterise microbial constituents within the prostate tissue of men with aggressive prostate cancer.

Previous studies have investigated the presence of bacterial, viral and prokaryotic organisms and their association with prostate cancer [9, 23, 24] using other methodologies including traditional bacterial culture, specific, targeted PCR and bacterial 16S rRNA amplification, traditional cloning and capillary sequencing methods. The advantage of MPS, in this context, is the capacity to sequence the entire genomic/transcriptomic content of samples without *a priori* knowledge of specific genes and targets [25], in addition to its sensitivity and high-throughput capability. However, despite the advantages of applying new technology to a decades-old question, the data generated and the methods used for data analysis were still in early development. As this field evolves, the methods, data, analytical tools and strategies will become more refined and enable further elucidation of these study questions.

To date, five studies [8, 9, 26–28] have investigated and characterised bacterial 16S rRNA sequences in prostate tissue collected from prostate cancer patients. Only one of these studies [28] found no evidence of 16S rRNA sequences in prostate cancer tissues. Four studies [8, 9, 26, 27] demonstrated the presence of bacterial sequences in 88.9, 85.7, 19.6 and 87% of patients, respectively. The most common organisms identified in these studies were members of the family *Enterobacteriaceae* and specifically species related to *Escherichia coli*. These findings are consistent with the results of the present study. In addition, analysis of the 16S rRNA V4 region sequencing data identified *Actinobacter* spp., *Pseudomonas* spp. and *Streptococcus* spp. as being present in 95% of all prostate samples therefore members of the ‘core’ community, in accordance with Sfanos et al. (2008). Analysis of the V2-V3 region also identified *Enterobacteriaceae*, *Escherichia* spp. as the predominant taxa within this sample of prostate tissues in addition to *Staphylococcus* spp, *Streptococcus* spp, *Moraxella* spp., and *Propionibacterium acnes* as members of the ‘core’ community.

Distinguishing between contamination of tissue and ‘true’ prostatic microbial constituents is one of the main challenges of bacterial community studies. Studies [8, 27] have suggested that the presence of bacterial sequences in prostate cancer tissues reflects bacterial contamination of the prostate via transrectal prostate biopsy of prostate which is routinely performed to confirm a diagnosis of prostate cancer. This could explain the presence of bacterial 16S rRNA sequences in prostate tissue samples from prostate cancer patients and the range of organisms detected in our dataset also supports this hypothesis.

Catheterization of patients has also been suggested as a way in which the prostate may be contaminated with bacteria. Hochrieter et al. (2000) detected 16S rRNA sequences in all four prostate tissue samples taken from a benign prostatic hyperplasia (BPH) patient that had an indwelling catheter for several weeks before radical prostatectomy [27]. Gorelick et al. (1988) performed quantitative bacterial culture of prostate tissues from prostatectomy patients to determine the prevalence of prostate bacterial infection or colonization [29]. They reported that 34% of patients with a pre-operative indwelling catheter returned a positive prostatic culture. Organisms were identified as common urinary tract pathogens including *E. coli* and *Streptococcus fecalis*. The pre-operative status with respect to catheterization of patients included in this study is unknown, however, it is a possibility that bacterial sequences identified in our samples could have been introduced in this way.

Sequences representing *Propionibacterium acnes* were detected in the V2-V3 16S rRNA dataset in 95% of samples albeit at low abundance. This study reports a 95% prevalence of *P. acnes* in prostate tissue samples which is consistent with the 100% prevelance of *P. acnes* detected in prostatic intraepithelial neoplasia (PIN) lesions and 78% of prostate cancer tissues reported by Fehri et al. (2011) but approximately two-fold higher than the prevalence of *P. acnes* reported by other studies [1, 2, 9, 30]. The present study could not determine whether the *P. acnes* sequences detected in the V2-V3 dataset represented either urogenital or cutaneous strains. Therefore, it is difficult to ascertain if the *P. acnes* detected in these samples represent contamination through laboratory handling and reagents or if they have biological significance.

The study design and methods employed in this study had several limitations that may have diminished the ability to detect infectious organisms in prostate tissues that were of clinical significance. The study design employed to identify potential infectious agents associated with prostate cancer was limited by study sample collection methods, the sampling of prostate tissue, small sample size and sensitivity of detection (total RNA sequencing). In addition, there were inherent limitations to our study design including the presence of multiple 16S rRNA gene copies, extraction methods, library preparation, experimental controls and bioinformatics approaches.

The 16S rRNA gene occurs in at least one copy of every bacterial genome, however can also occur as multiple and heterogeneous copies with copy number ranging from 1 to 15 [31]. The *E. coli* genome contains seven copies of the 16S rRNA gene and the *P. acnes* genome three copies [32]. Most 16S rRNA gene surveys assume that the relative abundance of 16S sequences are an accurate surrogate measure of the relative abundance of microorganisms in studies of community composition [31]. However, differences in the copy number/heterogeneity of the target 16S rRNA gene may result in overestimation of diversity and abundance [33, 34]. Therefore, inferences made on the basis of relative abundance of 16S rRNA genes may not be an accurate representation of actual community composition [31, 35] and variation in 16S rRNA gene copies can be a source of significant systemic bias within 16S rRNA gene surveys [33]. This study did not normalize for variation in 16S rRNA copy number and therefore it is unlikely that the reported relative abundances of taxa identified reflected *actual* taxa abundance. However, there are software tools [31] and a publicly available curated database (ribosomal RNA operon copy number database or rrnDB [35]) that could be applied to estimate actual organism abundance from 16S rRNA gene abundance data in future work.

There is considerable scope to extend and improve upon the experimental design of this study in investigating a *persistent* infectious aetiology for prostate cancer. Incorporating a prospective study design that collected tissues specifically for PCR- and sequencing-based analyses may reduce the prevalence of contaminating sequences. Inclusion of (a) control group(s) that included samples from lower grade and less aggressive prostate cancer cases and cancer-unaffected prostates such as those from organ donors, cystoprostatectomy and/or BPH cases would allow comparison between the microbial constituents of different prostate pathologies (if any) and normal prostate tissue. In addition, a greater number of cases would ensure that the study is sufficiently powered to detect differences in microbial communities (if any) between groups. Sampling a greater proportion of the prostate gland at several anatomical sites would provide comprehensive coverage of the prostate gland as a whole. With regard to 16S rRNA amplicon sequencing, the inclusion of extraction, PCR and water controls in sequencing runs would also provide a profile of laboratory contaminants so that ‘true’ microbial constituents (if any) could be distinguished from contaminating sequences. Normalization of 16S rRNA datasets to account for heterogeneity of 16S rRNA gene copies would also provide more accuracy with respect to relative organismal abundance. In terms of RNA sequencing, depletion of host RNA and enrichment of microbial rRNA and mRNA may increase detection sensitivity. If microorganisms of interest were detected, follow-up studies including verification of specific infectious agents in original nucleic acid samples via PCR and tissue localization studies would be warranted.

## Conclusions

An infectious aetiology for prostate cancer has long been conjectured. We evaluated new technology to assess if its use could clarify the inconsistency in evidence related to the nature of possible infection(s) and their relationship to prostate tumour grade. We applied targeted and agnostic approaches both involving MPS. This technology detected endogenous retroviruses providing proof of concept but there was no clear evidence of clinically significant bacterial or viral sequences in prostate cancer tissue. As these investigative methods and protocols become more refined, MPS approaches are anticipated to have significant utility in identifying potential pathogens involved in disease aetiology. Further studies, specifically designed to detect associations between the disease phenotype and aetiological agents, are required.

## Additional files

Additional file 1: Supplementary methods. (DOCX 21 kb)
Additional file 2: Complete 16 rRNA V4 region taxa summary. (DOCX 2335 kb)
Additional file 3: Complete 16 rRNA V2-V3 region taxa summary. (DOCX 1291 kb)

## Abbreviations

cDNA: complementary DNA
COX-2: Cyclooxygenase-2
DNA: Deoxyribonucleic acid
HERV: Human endogenous retrovirus
MPS: Massively parallel sequencing
mRNA: messenger RNA
OTU: Operational taxonomic unit
PCR: Polymerase chain reaction
RNA: Ribonucleic acid
RNASEL: Ribonuclease L
rRNA: Ribosomal RNA
SD: Standard deviation
TLR4: Toll-like receptor 4
