# Supplementary material for: Characterisation of microbial communities within aggressive prostate cancer tissues
Source: Infect Agent Cancer. 2017 Jan 13;12:4. doi: 10.1186/s13027-016-0112-7 (PMC5237345; doi:10.1186/s13027-016-0112-7)
Supplement: Additional file 1: — Supplementary methods. (DOCX 21 kb) [file 13027_2016_112_MOESM1_ESM.docx]

**Additional File 1: Supplementary Methods**

**Nucleic acid extraction**

Briefly, frozen tissue was initially disrupted by freeze fracture using the CryoPrep® (Covaris, Woburn, Massachusetts, USA). An amount of 600 μL of Buffer RLT Plus (Qiagen, Hilden, Germany) containing β-mercaptoethanol, was immediately added to the tissue, the tube capped and vortexed for 30 seconds. To further homogenise the tissue, the lysate was transferred directly to a QIAshredder® (Qiagen, Hilden, Germany) column and centrifuged at maximum speed for two minutes. The resulting lysate then underwent enzymatic digestion with proteinase K and incubated at 55^o^C for ten minutes. The sample was centrifuged at 10,000 g for three minutes and the supernatant transferred to a sterile 2 mL microcentrifuge tube. The sample DNA and RNA were separated by passing the supernatant through the AllPrep genomic DNA (gDNA) spin column (Qiagen, Hilden, Germany), which bound the sample DNA while the flow-though containing RNA was retained for extraction. The flow-though was transferred to a RNeasy® spin column (Qiagen, Hilden, Germany) and RNA extraction and purification was performed as per the manufacturer’s instructions with on-column DNase digestion. Genomic DNA purification was carried out according to the manufacturer’s instructions (Qiagen, Hilden, Germany).

**16S rRNA V4 hypervariable region primer sequences**

The primer sequences[11] are as follows:

515F- AATGATACGGCGACCACCGAGATCTACACTATGGTAATTGT**GTGCCAGCMGCCGCGGTAA** and

806R- CAAGCAGAAGACGGCATACGAGATNNNNNNNNNNNNAGTCAGTCAGCC**GGACTACHVGGGTWTCTAAT**.

The Illumina adaptors P5 and P7 are underlined in the 515F and 806R primer sequences respectively. The primer pad and primer linker for the 515F and 806R primer sequences are denoted by a dotted line and double underline while the broad-range primer sequences are in highlighted in bold. The index sequence is denoted by the series of Ns.

**16S rRNA V2-V3 hypervariable region primer sequences**

The primer sequences were modified from Caporaso et al. (2011)[11] using V2-V3 region specific primers[12] and are as follows:

101F- AATGATACGGCGACCACCGAGATCTACACTATGGTAATTGT**agyggcgiacgggtgagtaa** and

535R – CAAGCAGAAGACGGCATACGAGATNNNNNNNNNNNNAGTCAGTCAGCC**ATTACCGCGGCTGCTGG.**

The Illumina adaptors P5 and P7 are underlined in the 101F and 535R primer sequences respectively. The primer pad and primer linker for the 101F and 535R primer sequences are denoted by a dotted line and double underline while the broad-range primer sequences are in highlighted in bold. The index sequence is denoted by the series of Ns.

**16S rRNA sequencing primer sequences**

The primer sequences were as follows: Read1V4-TATGGTAATTGTGTGCCAGCMGCCGCGGTAA, Read2V4- AGTCAGTCAGCCGGACTACHVGGGTWTCTAAT, and IndexV4- ATTAGAWACCCBDGTAGTCCGGCTGACTGACT. The custom sequencing primers (adapted by Josef Wagner from the protocol of Caporaso *et al*. (2011)) for 16S rRNA V2-V3 region amplicon sequencing were as follows: Read1V2V3- TATGGTAATTGTagyggcgiacgggtgagtaa, Read2V2V3-AGTCAGTCAGCCATTACCGCGGCTGCTGG, IndexV2V2- CCAGCAGCCGCGGTAATGGCTGACTGACT.

**RNA library preparation**

In brief, RNA was fragmented by heat and divalent cations. The cleaved RNA fragments were then primed with random hexamers into first strand cDNA using SuperScript® II Reverse Transcriptase (Invitrogen™) and random primers. The RNA template was removed and a second cDNA strand was synthesised and marked by incorporating dUTP in place of dTTP to generate double-stranded blunt-ended cDNA. The 3’ ends of the blunt-ended fragments were adenylated with a single ‘A’ nucleotide to prevent chimera formation and also correspond to a complimentary single ‘T’ nucleotide on the 3’ end of the adaptor sequence that facilitates ligation of the fragment to the adaptor. Ligation of sequencing adaptors to cDNA fragments was performed. Enrichment PCR (13 cycles) of the first ‘unmarked’ cDNA strand was then carried out to selectively amplify only those cDNA fragments that had adaptors successfully ligated to both ends.
