# Supplementary material for: Characterisation of microbial communities within aggressive prostate cancer tissues
Source: Infect Agent Cancer. 2017 Jan 13;12:4. doi: 10.1186/s13027-016-0112-7 (PMC5237345; doi:10.1186/s13027-016-0112-7)
Supplement: Additional file 2: — Complete 16 rRNA V4 region taxa summary. (DOCX 2335 kb) [file 13027_2016_112_MOESM2_ESM.docx]

**Complete 16S rRNA V4 region taxa summary.** The contribution of taxa to each patient microbial community is expressed as a percentage. A – adjacent tissue, M – malignant tissue, k – kingdom, p – phylum, c –class, o –order, f – family, g – genus, s – species. Note: A coloured cell with a 0.0% figure indicates that the proportion is 0<x<0.1%.
